# IMPACT: A Structured Human-Centered Design Process for Integrating Community Health Worker Insights Into Program Policy in Pakistan

**DOI:** 10.9745/GHSP-D-24-00050

**Published:** 2026-08-10

**Authors:** Svea Closser, Marium Sultan, Erin Finley, Sue Gerber, Muhammad Shafique, Saeed Ahmed, Imtiaz Ali Shah, Naveed Khurshid, Farah Naz, Sadaf Nayyab, Celena Fei, Ali Sohail

**Affiliations:** a Johns Hopkins Bloomberg School of Public Health, Baltimore, MD, USA.; b The University of Texas Health Science Center at San Antonio, San Antonio, TX, USA.; c Independent Consultant, Truchas, NM, USA.; d Sohail Inc., Islamabad, Pakistan.; e Sohail Inc., Peshawar, Pakistan.; f Khyber Pakhtunkhwa Emergency Operations Center, Peshawar, Pakistan.; g World Health Organization, Peshawar, Pakistan.; h Johns Hopkins University, Baltimore, MD, USA.; i Sohail Inc., Karachi, Pakistan.

## Abstract

**Background:**

Community health workers (CHWs), most of whom are women, are the bedrock of primary health care provision in much of the world. CHWs are often employed at the bottom of health hierarchies where they have little voice; in state programs and vertical initiatives, accountability has generally flowed downwards. Yet many programs might function better if CHWs’ needs and ideas were considered in program design.

**Methods:**

From 2020-2022, we implemented a human-centered design process called IMPACT with CHWs working on polio vaccination in one district of Pakistan. The name IMPACT draws from the steps in the process: Identify problems and brainstorm innovations; Make and refine ideas; Present and evaluate ideas; and ACT to disseminate and implement. We held a facilitated competition for teams of CHWs, all of whom were women, to propose policy improvements to polio vaccination. In total, 417 CHWs participated in the design process facilitated by our team, and more than 500 additional CHWs participated in sessions facilitated by local supervisors. We worked with local policymakers to short-list the best ideas. Teams of CHWs presented the short-listed ideas to a panel of provincial and national-level policymakers, who selected ideas for implementation. We conducted interviews with CHWs and policymakers throughout the process to understand their experiences (n=82).

**Results:**

We received 181 idea submissions over two rounds of the process; 9 ideas were chosen for implementation. CHWs valued the process enormously; most said it was the first time their insights had been considered, and they wanted more opportunities for such input. The second round of the process was more effective than the first, with workers generating more complex ideas and program staff running workshops themselves. We heard, across management levels, that the process had positively impacted CHW motivation and confidence. Overall, the innovations selected for implementation were in the form of adjustments rather than major programmatic changes. Yet CHWs reported that the changes made were helpful and that it was very meaningful to have contributed to program policy.

**Conclusion:**

IMPACT facilitated substantive engagement and collaboration from both male supervisors and frontline female workers in a highly gender-stratified setting. Structured processes can allow the least powerful actors in global health interventions to draw on their frontline experience to suggest policy innovations.

## Abstract (precis)

A human-centered design competition successfully engaged community health workers in policy design to improve polio vaccination.

## Teaser

**Key Findings:**

- Structured human-centered design processes facilitated substantive engagement and collaboration from both male supervisors and frontline female health workers in a highly gender-stratified setting to propose policy improvements for polio vaccination.
- CHWs valued the process enormously; most said it was the first time their insights had been considered, and they wanted more opportunities for such input.

**Key Implication:**

- Structured processes can allow the least powerful actors in public health programs to contribute to improved program policy.

## BACKGROUND

Across the world, community health workers (CHWs) are the bedrock of primary care provision. CHW programs in low- and middle-income countries (LMICs) now comprise over 8 million workers.^1^ The majority of these workers are women, and many CHW programs in LMICs employ only women.

Such work is an important source of employment for women in contexts where other work may be unreliable or unavailable. Yet CHWs are frequently unpaid or underpaid.^2^^–^^4^ Furthermore, female empowerment through CHW work is complicated: although many women gain additional freedoms and power within the family, they are often employed at the bottom of health hierarchies where they are given little voice and even less opportunity to make suggestions for policy change.^5^^,^^6^ Especially given that CHW programs are frequently supposed to facilitate community participation in health programs, there is a need to find ways to actively empower CHWs within the programs that employ them.

Of course, inspiring examples of programs that have actively engaged and empowered CHWs as change agents exist.^7^^–^^10^ Most of these programs were designed from the ground up as activist projects. But in state programs and vertical initiatives that hire CHWs, accountability has generally flowed downwards, with CHWs’ work, timeliness, and behavior being closely scrutinized. Accountability, or even feedback, flowing upward—from CHWs to their managers or the health program itself—is much rarer.^10^

The World Health Organization (WHO), in their Global Health and Care Workers Compact, writes that health and care workers should be “engaged in the process of developing laws, regulations, and polices that affect them.”^11^ This certainly applies to CHWs, whose insights could lead to significant programmatic improvements. Many programs might function better if the needs of those actually implementing interventions were considered in program design.^12^^,^^13^

In this project, we asked: What opportunities for allowing CHWs to have additional voice and input in policy processes can be created in these programs? Through a human-centered design (HCD) competition we called IMPACT, we engaged CHWs as policymakers in polio eradication in Pakistan. The name IMPACT is an acronym drawn from the steps in the process: Identify problems and brainstorm innovations; Make and refine ideas; Present and evaluate ideas; and ACT to disseminate and implement.

## COMMUNITY HEALTH WORKERS IN POLIO ERADICATION

We approached CHWs working in a particularly challenging context: polio immunization campaigns in a polio-reservoir city in Pakistan. These areas have some of the highest rates of unvaccinated children in Pakistan and are underserved by services such as sanitation, water, and primary health care. The population overall is highly mobile, with a mix of people who have lived in the city for generations along with migrants from a range of settings. The social dynamics required to access individual households and address their needs are complex. Thus, while CHWs are assessed on their performance in this context, that performance is dependent on a number of factors that are in many cases beyond the CHWs’ power to influence.

The work is not only difficult but also dangerous: many frontline workers have been murdered working in this job.^14^^,^^15^ It is also critical, as the global eradication of polio hinges on high vaccination coverage in these areas. It was in this context that we worked with CHWs to bring their expertise and insights into policy improvements for vaccination coverage.

In polio-endemic areas, the Pakistan Polio Eradication Initiative (PEI) employs paid full-time staff separate from the Ministry of Health; these CHWs are responsible for vaccinating children against polio, with the aim of eliminating the disease.^16^ In a striking and valuable move unusual for global health agencies and governments, who generally employ volunteer or per-diem staff, these women are paid at or above minimum wage. This wage can provide a lifeline to vulnerable women, many of whom feel a deep commitment to their jobs in ways that are not common in volunteer or low-paid CHW programs.^17^^–^^19^

The Polio Eradication Independent Monitoring Board, in its 17th report, urged development of a “supportive, empowering, problem-solving performance culture for the front line” and encouraged solicitation of feedback from frontline staff on practical difficulties and morale.^20^ Additionally, the Independent Monitoring Board recommended that “improving communication—in particular, starting with sensitive listening—should be at the heart of the polio programme at every level.”

**PHOTO:**
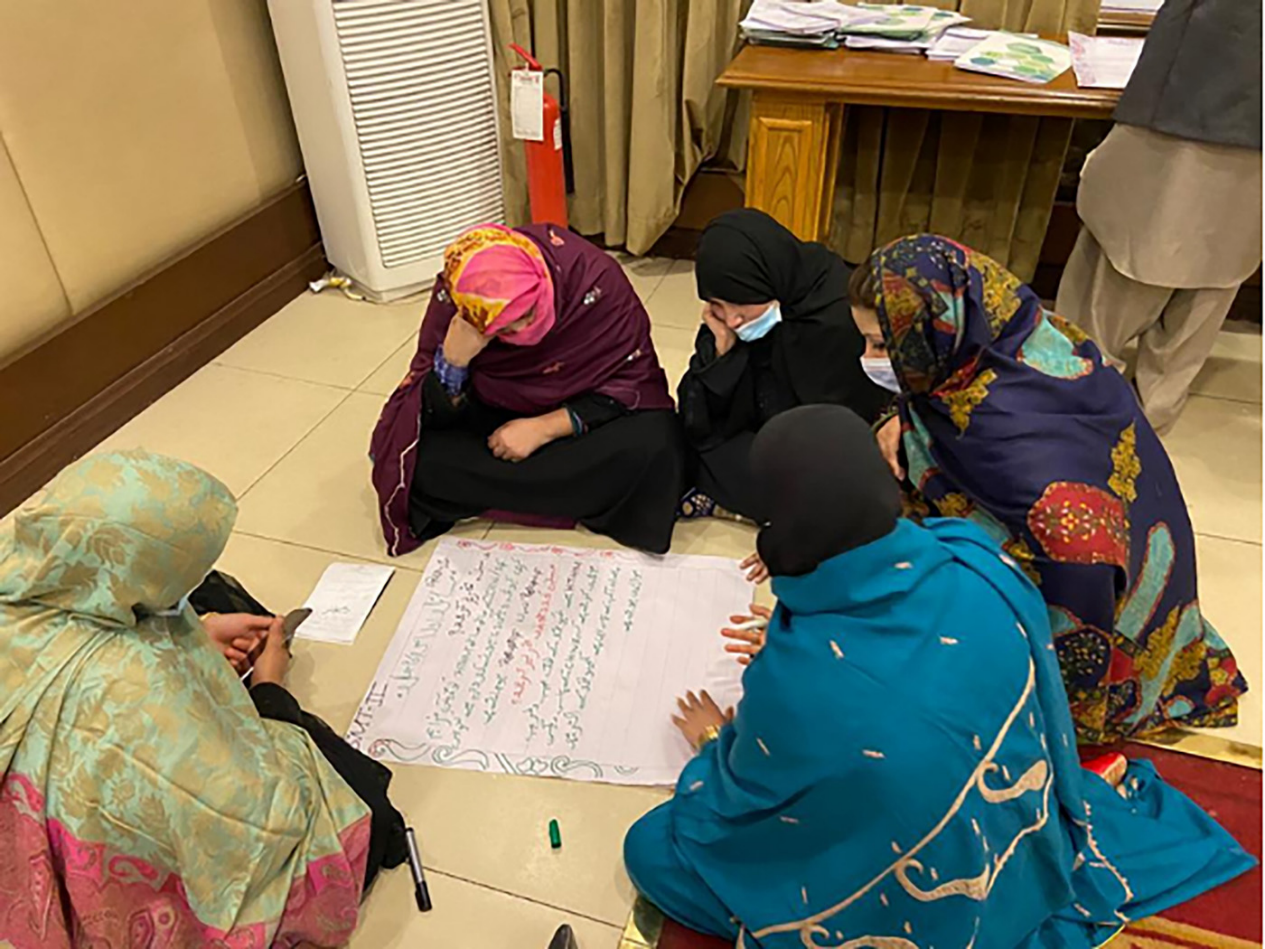
Photo caption and credit: During a brainstorming session, community health workers prepare a poster describing their idea to improve polio vaccination. Photo: Sohail Inc., 2021.

## DESIGNING THE IMPACT PROCESS

We designed this intervention to respond to these recommendations. We developed the IMPACT process through discussions between Pakistan PEI stakeholders and external academic and implementing partners. The goals of the project were to:

1. Work with CHWs to develop and refine ideas for program improvement
2. Disseminate leadership-approved innovations across the system

Our ideas were that CHWs’ field experience meant they had an excellent vantage point to create effective, context-appropriate strategies, and that engaging CHWs in program design could give them a new ownership over and commitment to their work.

Through the IMPACT process, we engaged CHWs in persistently polio-endemic areas, called Super High-Risk Union Councils (SHRUCs), in one district in Pakistan. This district has ongoing operational and community engagement challenges we hoped CHWs could help address. In SHRUCs, the PEI adopts a vertical approach with paid full-time CHWs contracted through PEI partners rather than engaging government CHWs.

The IMPACT process is an adaptation of a “Shark Tank” competition process, which has proven effective at integrating frontline worker insight into program design in the United States.^21^^–^^24^ The process drew on HCD principles but adapted and extended the traditional HCD process to include processes from dissemination and implementation science aimed at institutional dissemination and sustainment.^24^^,^^25^ We chose specifically to adapt the Shark Tank process—rather than other “bottom-up” policy processes—because it included both frontline worker design and structured engagement with higher-level officials that could facilitate adoption of CHW-designed policy changes across the system.^22^ We made major modifications to this process to tailor it to the very different context of polio eradication in Pakistan, with an eye to providing a forum where CHW insights could be listened to and taken up by program administrators.

## METHODS FOR EVALUATING THE PROCESS

To get participant feedback on our process, we conducted 3 rounds of interviews with frontline workers, managers, and higher-level stakeholders involved with IMPACT. These interviews were conducted in Urdu, Pashto, or English, depending on the preference of the interviewee, by interviewers who were native speakers of these languages. Interviews with CHWs were in person and conducted by women; interviews with managers and higher-level stakeholders were conducted by male and female interviewers either in person or over Zoom.

The first round of interviews took place before we began the competition in 2020 and helped inform our process; the second between the first and second rounds of our competition in July 2021, which helped us refine our methods; and the third at the end of the project in late 2021, inviting participants to reflect on the successes and challenges of the process. In this article, we refer to these as baseline, wrap-up, and endline interviews, respectively. We spoke with 63 polio CHWs, managers, and higher-level officials at baseline; 25 during wrap-up; and 28 at endline. In total, we interviewed 82 people about the program, some of them multiple times. While the baseline interviews focused on work experiences and challenges faced by CHWs in the polio program, the wrap-up and endline interviews focused on the IMPACT process itself, asking participants for their feedback on the process and their suggestions for improvement. A description of the interview guide for the baseline interviews, with guidance for adapting it for other programs, is on pp. 10–12 of the IMPACT Toolkit included in the Supplement.

To better understand the context of polio work, members of our team participated in the polio campaign in June 2021. We accompanied polio workers, at the frontline and management levels, in their daily activities, including door-to-door vaccination, data collection, social mobilization, and data compilation.

Two female team members carried out participant observation with female frontline workers; both team members were local to the district and familiar with life in the SHRUCs. Two male team members conducted participant observation in the male-dominated spaces of the Union Council and city leadership. The project team worked together to construct field notes from this participant observation. These same team members facilitated the brainstorming sessions in the IMPACT process, meaning that their facilitation was enhanced by the knowledge they had gained from participant observation, as well as their personal relationships with many CHWs.

All interviews were transcribed and translated into English, and interviews and field notes were entered into the analysis program MAXQDA. A team of analysts familiar with the context of polio work in Pakistan coded interviews and field notes, using inductive coding to capture the categories emerging from the data.

In addition to the qualitative work, we had initially planned a quantitative analysis of the effects of the program. We planned to compare coverage numbers with previous campaigns, and also compare coverage in the same campaign in areas where the CHW-generated innovations were and were not implemented. However, neither of these comparisons proved possible. As this project was implemented in the midst of the COVID-19 pandemic, constant changes in COVID-related restrictions and protocols meant that coverage rates in one immunization campaign were not comparable with those in previous campaigns, making before–after comparisons meaningless for the purposes of evaluating our project. Further, it did not prove possible to implement CHW-generated innovations in only some areas and not others to test impact. For example, one CHW-generated innovation was implementing changes in the length of CHW employment contracts, something which was necessarily done at the national level for all CHWs. In the end, we prioritized programmatic goals over evaluation goals and did not conduct the quantitative analysis.

Here, we describe each of the 4 main steps in the IMPACT process and include qualitative information on what our interviewees said about the process at each step. Our goal is to provide sufficient information to allow others to adapt the process to other contexts where it may be useful. More detailed information on the process is also available in the IMPACT Toolkit, included in the Supplement.

## THE IMPACT PROCESS

**FIGURE 1:**
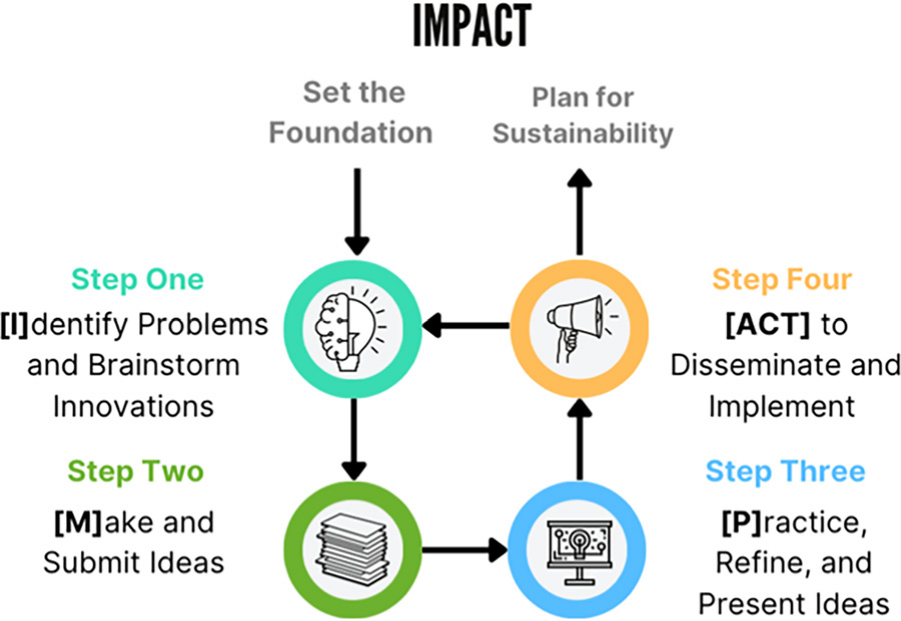
IMPACT Process Steps

The IMPACT process consists of 4 steps, which we describe in turn here (Figure 1). First, groups of CHWs self-identify the problems they face and brainstorm innovative solutions to those problems. Second, they submit their ideas for solutions to the IMPACT competition. Next, IMPACT competition finalists are selected from the groups of CHWs that submitted ideas. Those groups are notified that they are finalists and supported in practicing and refining pitch presentations, which they give to a panel of health policymakers. Those policymakers choose CHW ideas for implementation. CHWs are rewarded for their innovation and the selected ideas are implemented.

### Setting the Foundation

**FIGURE 2:**
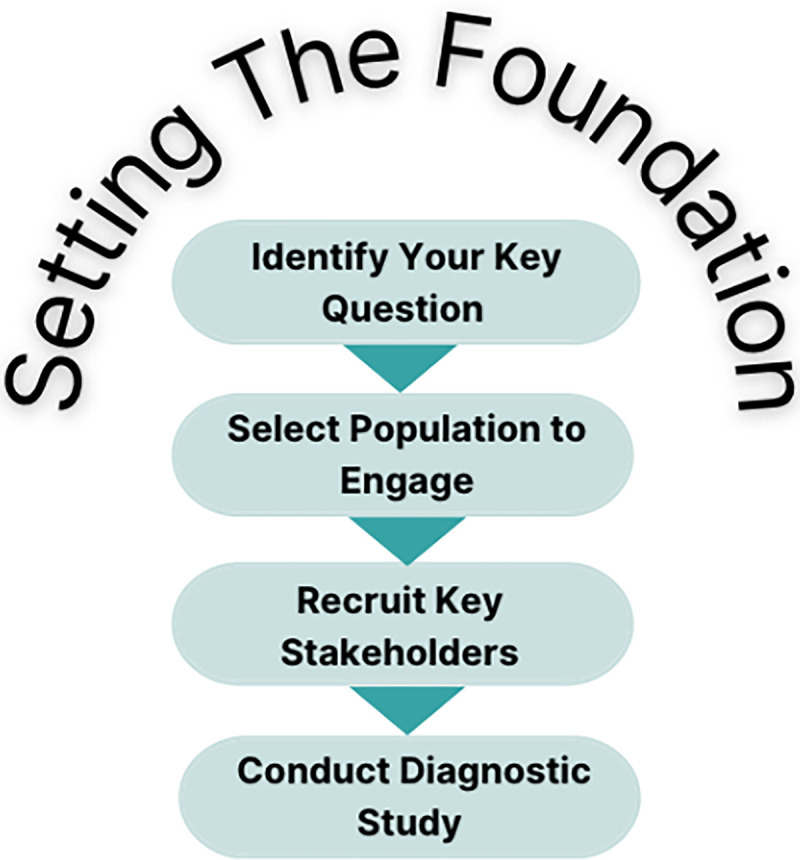
Key Foundational Activities in the IMPACT Process

Before beginning the main IMPACT competition process, we set the foundation for the work by identifying the aim of the process; selecting the population to engage; recruiting key stakeholders; and conducting a diagnostic study (Figure 2).

#### Identifying the key question

We started by establishing clarity on the health service for which we were asking CHWs to design policy. In the case of our project, we asked for ways to improve oral polio vaccination coverage in door-to-door campaigns. This was specific enough to be very clear while also being broad enough to give competition participants wide leeway in the kinds of problems they would target and solutions they would propose. They could, and did, suggest policy changes to address concerns as wide-ranging as vaccine hesitancy, human resource issues affecting health workers, challenging gender dynamics, and logistics of vaccination timing.^26^

#### Selecting the population to engage

We decided to work with CHWs because we felt they had great insights from their day-to-day work that were perhaps underappreciated in program design. The district where we did our work was chosen by provincial officials because of its challenges with community engagement and program implementation.

During the first round, we selected around half of the total SHURCs in our target district to conduct the IMPACT process. This sample of Union Councils was selected for (1) having at least 20 CHWs; (2) including SHRUCs with high polio vaccine coverage and those with low polio vaccine coverage; and (3) geographical proximity, making project management feasible. We engaged 261 CHWs in our competition in the first round. After this first round, we refined our process, and in the second round we expanded IMPACT to all SHRUCs in the district. In the second round, we engaged 156 CHWs directly; in addition, we also trained local supervisors to run the sessions, and those sessions run by local staff engaged more than 500 additional CHWs in the competition. To protect CHW anonymity in this article, we are not specifying the district. All CHWs of SHRUCs in which we conducted IMPACT were invited to participate in the process.

#### Recruiting key stakeholders

The PEI structure in Pakistan includes many institutional actors, including stakeholders from the national, provincial, and district governments; WHO and UNICEF staff at the national, provincial, and district level; and additional partners such as the IMPACT staff for this project. These actors come together at Emergency Operations Centers and Emergency Response Units at the national, provincial, and district levels in areas of Pakistan at high risk for polio, including the city where we were working. It was critical for us to work closely with people from the Emergency Operations Centers at all these levels, as they would be the ones selecting CHW-generated ideas for implementation and ultimately implementing them. The dedicated IMPACT team could facilitate, but not drive, the process.

As described above, the IMPACT process was developed collaboratively with PEI stakeholders at national and international levels, but before beginning the program we tailored the process through discussions with district- and provincial-level officials. In a project aiming not just to get input from CHWs but also to see their policy ideas through to implementation, this was a critical step. We held meetings with local, regional, and national policymakers to explain the process, get their input on how to structure it, and get their commitment to participating in the competition and supporting the policy changes that emerged from it. The response was generally supportive; although some people we spoke to were reasonably skeptical that CHWs would come up with programmatic ideas that had not occurred to anyone else in the program in its 25-year history, nearly everyone felt that more engagement and support for CHWs—who were, people agreed, doing very difficult work—was a good thing.

#### Conducting a diagnostic study

Before we began the IMPACT process, we conducted the baseline interviews described above with CHWs and their managers to gain an understanding of the challenges faced by polio CHWs and the program more broadly. This work provided us with a broad understanding of the issues faced by CHWs in their work, enabled us to be informed facilitators of the IMPACT process, and helped us build relationships with CHWs that improved the IMPACT process.

### Step 1: [I]dentify Problems and Brainstorm Innovations

Step 1 of the IMPACT process is an ideation process grounded in HCD principles; however, we adapted the usual steps of the HCD process, as outlined by IDEO, based on input from our experienced local staff regarding what would work best for this context and program.^27^ The first step of the IMPACT process was to have teams of polio CHWs identify problems they faced in effectively conducting their work and to brainstorm innovative solutions to those problems. We held day-long brainstorming workshops in which groups of 6–8 CHWs who worked together on a regular basis teamed up to select a problem to solve and to brainstorm solutions to that problem. CHWs’ participation in these workshops was covered by their salaries, and we also provided a per diem to cover transportation costs.

The day-long brainstorming workshop started with an activity-based icebreaker, followed by introduction of the IMPACT process and discussion on the rationale and purpose of the workshops. Teams started by identifying problems or issues they faced in their work. This was followed by a problem unbundling exercise, where CHWs learned to dig into the root causes behind the issues they had identified utilizing the 5 Ws approach (who, what, when, where, why). Each group then chose at least one problem to focus on for the rest of the day.

### Step 2: [M]ake and Submit Ideas

**FIGURE 3:**
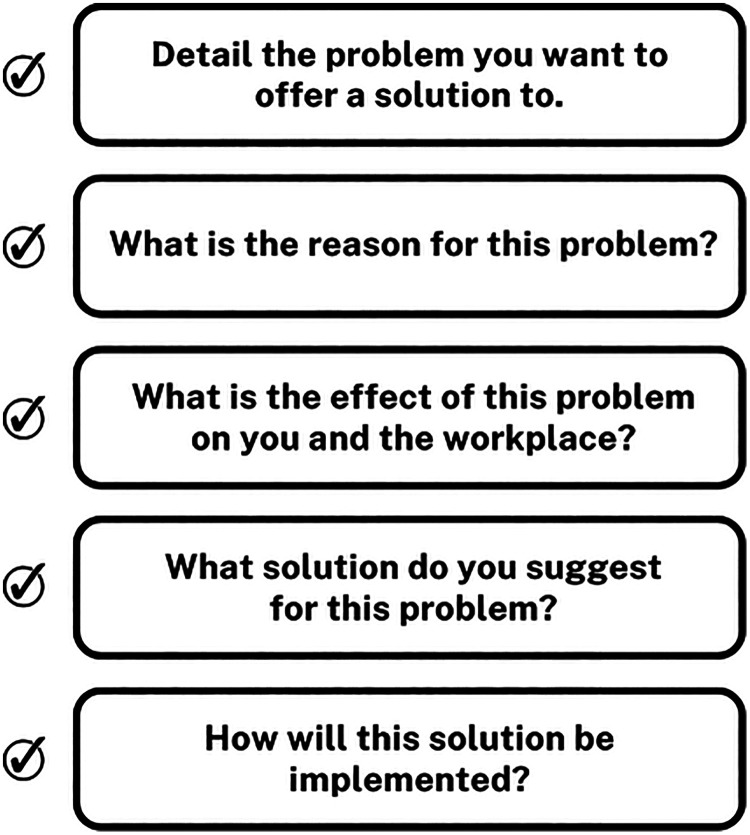
Question Guide to Help CHWs Develop Ideas to Improve Polio Vaccination

Teams spent the second half of the day coming up with a solution to at least one problem they had identified. After we walked teams through some hypothetical examples, they worked independently, filling out a 5-page form with one question on each page (Figure 3). At the end of these day-long workshops, each team submitted at least one idea. Some teams submitted additional ideas after completion of the workshop.

#### Collecting and summarizing ideas

Over the course of two rounds of IMPACT, we received 181 idea submissions, each generated by a group of CHWs. In each round, we synthesized and summarized the problems and solutions from the handwritten documents, submitted in Urdu, onto an Excel sheet in English. We grouped similar topics together to help streamline the evaluation process. Further information on collecting and summarizing materials is on p. 20 of the IMPACT Toolkit in the Supplement.

#### Creating a long list of good ideas

We excluded ideas from consideration if they were already being implemented in the program or if they were related to an issue with an individual person rather than applicable to polio eradication implementation or policy more broadly. The ideas that remained were scored on a rubric by 4 PEI reviewers from the provincial level, 2 PEI reviewers the district level, and 4 members from IMPACT team.

The rubric considered the relevance and severity of the problem that was identified; the clarity of the solution presented; the ease of implementation of the solution, in terms of both time and finance; the likelihood that implementing the idea would solve the problem; and the sustainability of the idea (Box 1). Given the wide variation of types of ideas we received, we allowed these reviewers to use their experience and judgment in scoring each idea according to these broad criteria. The highest scoring ideas were moved to a “long list” for in-person discussion.

BOX 1 Scoring Rubric to Move CHW-Generated Ideas to Long List for Consideration

**Filtering Criteria (if either are yes, the idea does not proceed)**

- The idea is currently in the works of implementation (Y/N)
- The idea is related to an individual rather than applicable to the team or Polio Eradication Initiative (PEI) (Y/N)

**Weighted Criteria (score 1–5 for each)**

- Relevance and severity of the problem for PEI (20% weight)
- Clarity of idea (5% weight)
- Ease of implementation in terms of time (20% weight)
- Ease of implementation in terms of finance (10% weight)
- Likely potential of the idea to solve the problem/potential of impact (25% weight)
- Value for money (10% weight)
- Sustainability (10% weight)

#### Creating a short list of teams to move to the pitch presentation phase

Next, we convened district and provincial officials in an in-person meeting to discuss which of the long-listed ideas should be short-listed, which meant that the teams of CHWs who created those ideas would be invited to present their ideas in a pitch presentation to polio leadership. Members of this panel, most of whom had participated in the scoring process, were often very passionate in advocating for particular ideas to be short-listed, based on their own experiences of issues in the field. The IMPACT team served as moderators through this process. At the end of the meeting, we decided on 5–10 ideas to be presented by representatives of their respective team through pitch presentations.

### Steps 1 and 2: Considerations and Suggestions for Success

#### CHW feedback

When we interviewed them later, CHWs were very enthusiastic about the brainstorming workshop process. Many said it opened a beneficial new way of relating to their work. “Even when we went home after the workshop,” one commented, “we started thinking about other issues that we could work on.”

Many CHWs said that this was the first time they had been given the opportunity to discuss their problems and give ideas for how to solve them. One commented, “We are happy that there was someone who considered us part of the polio program.”

Another added, “For the first time, we had found someone who wanted to listen to our problems and suggestions, and we hoped to solve the problems and implement our ideas.”

These comments were borne out by our observations. In the first round of our process, only one CHW failed to show up for the brainstorming session; she had just broken her leg and was reportedly in tears over the fact that she could not attend. At the end of many of the sessions, CHWs held impromptu celebrations in the workshop venue (male facilitators were asked to leave but female facilitators were invited to stay). We simply have never had more enthusiastic participants in any CHW project in our careers.

#### Teamwork

CHWs in this project said they enjoyed the teamwork aspect inherent to the process. They felt that solving problems with the same group of women they worked with on a regular basis brought them closer together. One commented:

*We worked with the team. That experience was wonderful too. Because we had the opportunity to learn from each other and to put our opinions in front of each other. We corrected each other and learned a lot from each other.*

The groups we created included CHWs as well as their female immediate supervisors. We were aware that this hierarchy was likely to affect group dynamics, and we did our best to position CHWs and their immediate supervisors as equal participants in the process, encouraging equal voice through round tables and icebreaker exercises. CHWs told us they valued this part of the process. One commented:

*We were given freedom and equality. There was no concept of senior or junior, we all worked on everything together as one team.*

Of course, hierarchies did not disappear entirely. When teams were chosen as finalists and worked to prepare pitch presentations, supervisors tended to have more visible roles.

#### Workshop leaders

During the first round, our team led all the brainstorming sessions. The second time we ran the competition, we trained district-level managers to lead the sessions. They collected the ideas and passed them on to us.

We had initially assumed that manager-led sessions would lead to less engaged CHWs. During interviews with CHWs, we heard many felt their continued livelihood was contingent on appeasing their seniors. However, we soon learned that manager-led sessions worked as well as or better than sessions led by our team of facilitators; we received a similar quality and variety of responses across both types of sessions, and CHWs in both types of sessions identified issues with supervisory structures.

#### Venue

The venue of the sessions mattered to CHWs. During the first round of the competition, in order to demonstrate to CHWs how much they were valued, we chose to rent a conference room in the most upscale hotel in the city for our brainstorming sessions.

A manager described how this made CHWs feel respected and honored:

*One thing I want to mention is that the environment that you had created and the trainers you had invited was in itself a motivation for them. … If a person is going to a VIP place, they feel it an honor for themselves, they feel that God is rewarding them for their work. So your venue was the first strength of brainstorming, their feedback was very positive too.*

CHWs described how proud they were to be attending an event at this hotel. One said that her rickshaw driver did not believe this was the destination she was going to, as someone of her socioeconomic status would not usually have the chance to enter into that space:

*First of all, we thank Allah that we were also invited to [this] hotel, because what is our status? … We were proud of our education and we were happy that you gave us this opportunity.*

However, the location was hard to reach by public transit and, at the suggestion of a manager, our second-round workshops were conducted at a less upscale but more accessible venue. There were mixed feelings about this change on the part of CHWs and supervisors. We suggest that if funding is available, it is likely worth the investment to choose an upscale location for these sessions, to convey to the participants that they are valued.

#### Multiple teams with the same idea

Many ideas were received in the same or similar form from multiple groups of CHWs. In preparing the summary spreadsheet, we linked teams with the same central concept. Grouping ideas helped us understand how many frontline workers supported any given solution and simplified the voting process.

However, this led to challenges in terms of determining a way to reduce the number of short-listed CHWs we would engage with. For example, in one round of our competition, 8 ideas were short-listed. These 8 ideas had been submitted by a total of 22 teams—too many to engage in a pitch presentation day.

We managed this by combining teams in the pitch presentation process, aiming to engage a group of 8–10 CHWs per idea. If a short-listed idea had been submitted by just one team, all members of that team were invited to participate in the pitch process, but if a single idea had been proposed by 8 teams, each team was asked to nominate one person to participate in the pitch process. Regardless of whether they were included in the pitch process, all members of all short-listed teams received an individual cash prize. These cash prizes were significant—about one month’s salary—and delivered directly into CHWs’ bank accounts by the payroll vendor.

#### Scoring ideas—who and how

Our short-listing and long-listing process was driven largely by program leadership at district and provincial levels. In future iterations of this project, we might include CHWs on the scoring team. That said, it was important that the people who would ultimately be responsible for implementing the selected ideas had a central role in the selection process, both because they understood best what was institutionally possible and because in this context, nothing would be implemented without their buy-in. People scoring the ideas had not been directly involved in the ideation sessions, and they could not identify which teams had submitted which ideas.

From the perspective of the IMPACT team, it was not always the “best,” most innovative, or creative ideas that were short-listed, but the ones that were seen as most valuable by program leadership. Since winning ideas ultimately need to be implemented by the program itself, we believe this process was essential in seeing ideas through to implementation. For policymakers to shepherd an idea through to full implementation, they needed to be excited about the potential of the idea.

### Step 3: [P]ractice, Refine, and Present Ideas

In addition to providing all members of teams whose ideas were selected for pitch day with a cash prize, we also held a certificate ceremony recognizing these teams. Representatives of the teams whose ideas were short-listed for the pitch day were invited to meet to refine and prepare their ideas.

#### Refining the ideas

Each of the short-listed teams participated in a half-day session with IMPACT staff. In this session, the teams discussed and refined their ideas, prepared presentation slides with the support of the IMPACT team, nominated 2 presenters, and practiced their presentations.

During these sessions, we conducted activities to help prepare the short-listed teams for pitch day. We invited them to imagine what the program would look like if the problem they described was solved and to communicate that to their audience. We also did an activity in which we acted as a devil’s advocate, encouraging them to think through convincing answers to tough questions. Although we facilitated their process of refining and promoting their ideas, we did not direct them what to propose.

#### Practicing the presentations

Prior to pitch day, we conducted a 4-hour rehearsal session with all short-listed teams. Two team members from each team presented the pitch for their group to the larger group. Other CHWs and the IMPACT team asked questions to the presenting team and gave feedback where needed.

#### Holding the pitch day

As the final step to select winning ideas, CHWs presented their ideas to a panel of polio leadership in a formal pitch day. Panel members asked questions and engaged in discussion with CHWs on the team after each pitch. After hearing all the pitches, the panel convened to choose 4–5 winning ideas to go forward for implementation.

During Round 1 of our process, facilitated by our IMPACT staff, we held brainstorming sessions with 39 teams, and 5 ideas were selected for implementation. Round 2, 9 months later, was facilitated by program staff who had been trained in the IMPACT process. During that round, we received ideas from 142 teams; 4 were selected for implementation.

Most of the solutions proposed by CHWs across the 2 rounds fell into 4 themes: reducing community fatigue, strengthening primary health care, improving community engagement, and workplace issues (Table). Across ideas, we heard that the broader provision of primary health care was key to community acceptance of intense and repeated vaccination campaigns and that workplace supports for CHWs were critical. More details on these ideas is available in a separate paper.^26^

**TABLE.** Community Health Workers’ Ideas to Improve Oral Polio Vaccination, by Theme

	**No. of Teams Submitting Idea**
**Tackle Community Fatigue**	**35**
Hold fewer campaigns and fewer household visits per campaign	21
Streamline data collection and improve data management	6
Other	8
**Strengthen Primary Health Care**	**39**
Provide health camps	6
Improve government clinics	23
Improve water and sanitation	1
Implement co-delivery of additional services during polio campaigns	9
**Engage the Community**	**38**
Engage religious influencers	7
Manage refusals by doctors and government workers	5
Introduce educational videos	7
Create a polio dial tone	3
Other	16
**Improve the Workplace**	**77**
Reduce overtime work	10
Improve processes for receiving workplans	5
Hire more selectively	10
Enhance skills training	5
Implement longer-term contracts	7
Create space to discuss workplace issues	4
Address violence from the community	5
Other	31

### Step 4: [ACT] to Disseminate and Implement the Best Processes and Ideas

The IMPACT process would, of course, be incomplete without the implementation of the winning ideas (Box 2). By selecting ideas for implementation on pitch day, the selection panel of policymakers committed to implementing those ideas. That said, we found it helpful to have our team support the process of implementation.

BOX 2 CHW Ideas for Improving Oral Polio Vaccination Selected for Implementation

**Round 1 Winning Ideas:**

- **Respect Working Hours:** CHWs should not be asked to respond to routine data requests outside working hours.
- **Implement Capacity Building:** CHWs should be trained on responding effectively to vaccine refusals and conspiracy theories.
- **Clearly Define CHW Roles for Routine Immunization:** Polio CHWs should have clearly defined roles and streamlined touchpoints for supporting routine immunization.
- **Increase the Duration of CHW Contracts:** CHWs should have contracts of longer than 3 months.
- **Address Vaccine Refusals by Government Staff:** Vaccine refusals by medical professionals and government employees should be addressed through engagement in government structures.

**Round 2 Winning Ideas:**

- **Provide Recognition for CHWs:** High-performing CHWs should be rewarded for excellent work.
- **Provide Consistent Instructions:** Directives from different supervisory levels should be harmonized so that CHWs have clear and consistent work instructions.
- **Implement a Polio Ringtone:** Special polio ringtones should be used to help increase coverage of vaccination awareness messages.
- **Hire Female Religious Influencers:** Women with religious affiliations should be engaged to speak to women with religious concerns about vaccination.

Our team worked with polio leadership to identify individuals responsible for implementation of each of the selected ideas, and we further worked to develop an implementation plan for those ideas and to monitor and support progress in implementation. We also served as a bridge between the implementation team and the policymakers who had selected the idea, keeping policymakers updated on challenges in the implementation process and requesting their support where necessary.

The trajectory of implementation of the winning ideas varied. Some were implemented fully; for example, a winning idea that CHWs should not be subject to data requests outside of working hours was given strong backing by district polio officials, and our observations and interviews bore out that this change happened. Other ideas were implemented partially; for example, a winning idea regarding training in community engagement resulted in some changes to training protocols, if not everything that CHWs had suggested. Some other ideas were not ultimately implemented; for example, a winning idea that government employees should be approached by their supervisors if they refused vaccination proved too politically challenging to implement in practice.

### Steps 3 and 4: Considerations and Suggestions for Success

#### CHW feedback

CHWs told us that one of the best points of IMPACT was being able to speak directly to senior program officials. One CHW who participated in a pitch presentation commented:

*We were called to represent our ideas in front of higher authorities. They really liked our idea and encouraged us. What we are really happy about is that officers in such high posts gave our views recognition. It was a very fruitful experience.*

Another CHW commented, “… after getting appreciated for our idea, we were really content.”

There were practical benefits as well. One CHW said:

*This workshop resolved our problem [by implementing our idea], and because of that now our vaccination coverage will be 100% instead of 80%. We are really thankful that they selected our idea and solved the issue for us.*

#### Involvement of policymakers at different levels of the system

We found it very useful to have engaged officials at the highest levels of the program from the outset of our process. This was key in giving us legitimacy as an actor in the system and helped us initially make connections with policymakers at provincial and district levels.

Although we endeavored to engage national-level policymakers in our process, we found district and provincial leadership were more engaged and active in our day-to-day work. They understood our process and the ideas that were being presented fully, and working with them was very smooth. We did need to engage national-level policymakers again in the process of implementation for a few ideas, such as changes in contracting processes for CHWs.

## PLANNING FOR SUSTAINABILITY

Throughout the process, we kept the district-level administration closely involved and up to date. Over time, they gained a strong understanding of the IMPACT process. They told us that they were fully convinced of the value of reaching out to the frontline for ideas, and they appreciated how the process motivated frontline staff. They ran workshops in Round 2 very successfully, and they were clear that they wanted to continue the process in some form after our project ended.

The tricky part of sustainability, then, was not conducting the workshops themselves. Nor was it buy-in from leadership. Rather, it was finding staff who, in the context of an eradication push, had time to read all the CHW-generated ideas, collate them, organize feedback, train teams for pitch presentations, and hold the pitch day. This is dedicated full-time work for 1–2 people for several months per round, and district leadership did not have the bandwidth to do this in addition to all their routine tasks.

After our project ended, the polio program in Pakistan saw sufficient value in the project that it customized it to scale it as a problem-solving medium at the national level, holding workshops with CHWs across the country. They hired members of the original IMPACT team to facilitate this process. In addition, the program continues to hold what it now calls “listening sessions” with CHWs to get their input.

The program, then, has found ways to integrate the spirit of this process into its programming, in a less labor-intensive way. To truly sustain this program in its full form would require the institutionalization of the kinds of work the IMPACT team was doing in formal programmatic job requirements. For example, CHW trainers or training institutions might have this process included as part of their contracts.

Officials within the polio program told us that they found the workshop model useful for two reasons. First, it allowed them to efficiently get feedback and insight from CHWs into frontline conditions and operations. Second, it was very motivating for the CHWs who participated in it, which higher-level officials felt was beneficial to the program as a whole.

## FEEDBACK FROM CHWS AND POLICYMAKERS

The feedback we got from participants and policymakers is helpful in thinking about using this process in other contexts.

### Listening up the Ladder

Generally, the only communication between the CHWs and higher-level provincial, national, or international officials was through the data they generated on topics such as immunization rates and reasons for vaccine refusal. While this data is of course important, CHWs commented that they had many other insights and were happy to be able to share them and contribute to policy. Many CHWs highlighted that this was the first time they were asked their opinions on how the program was run.

“We know about the gaps and issues in the program which are realized in the field,” one CHW commented. “This was the first time that someone had asked for our suggestions.”

This made CHWs quite motivated to participate. One CHW said she participated “willingly and eagerly.” Another commented:

*We participated in this program of our own desire because it was an opportunity for us to share our problems. Our monitors do not listen to our problems in the field, so that’s why we felt really good about participating in this workshop.*

Through the process, the value of CHW insight also became clear to leadership. In the first round of pitch presentations, a few people in high-level leadership at first expressed doubt that disadvantaged CHWs could have such insightful commentary into the complexity of the challenges they faced—implying that perhaps our team had in fact created the pitch presentations. However, as these leaders asked probing questions and got detailed and thoughtful responses from CHWs, they became convinced of the depth of CHW insight, and these concerns evaporated. By the second round of pitch presentations, leadership was prepared to see CHWs as experts.

“Something that really made us happy,” one CHW commented, “was that our views are being heard and respected by senior leadership.”

A few CHWs said this experience had boosted their confidence in their day-to-day work. “We can now talk to our seniors without hesitation or fear,” one CHW said, “and can communicate our issues with them.”

District-level officials also told us these new lines of communication were valuable. These officials commented that in the normal course of their fieldwork, CHWs did not generally open up to them about their issues. They commented that they had never heard a lot of what came up in the ideation process, and they would like to continue to keep these lines of communication open.

### Ensuring Friendly Facilitation

Part of listening was having facilitation in the workshops that aimed not to over-direct the process. We made sure to emphasize that the facilitators were there to hear CHW ideas and that the CHWs were the experts. We spent time on engaging, interactive icebreakers at the beginning of our workshops. We paid a great deal of attention to how facilitators approached the process, and CHWs said they appreciated that orientation. One CHW commented:

*Whenever we didn’t understand, the trainer [facilitator] came to our table and guided us. It was a very friendly atmosphere, and we didn’t feel that they are our seniors, they were just like our friends.*

### Recognition for Winning Teams

The impact of being heard and respected was particularly significant for winning teams. “They told us that our idea got selected from among 156 teams,” one CHW commented. “That was a moment of pride for us. Our colleagues were pretty happy with the whole process.”

We were initially concerned that the competition might create resentments within cadres of workers, but this did not prove to be the case. Rather, expectations were sufficiently low at the outset regarding the outcomes of this process that the fact that *any* CHWs were rewarded was a positive surprise.

CHWs said that one of the biggest benefits of the workshops was feeling appreciated and that this was well worth the extra time they put into the process. “The selection of our idea was even more meaningful than getting money from the IMPACT team,” one winning team member commented.

### The Value of Continuous Engagement

In both the wrap-up and endline interviews, we heard from CHWs that they had more ideas they wanted to share and that they wanted to continue to have the opportunity to provide those ideas. Several CHWs commented that after the workshops, more ideas came to them. “Even when we went home after the workshop,” one commented, “we started thinking about other issues that we can work on.”

“There should be more workshops like this,” another commented. “Because over time, more suggestions come to mind about improvements to the program.”

Some managers also commented that they saw the benefit of IMPACT on CHWs and that holding such sessions every few months would be valuable.

### Giving IMPACT Opportunities to More Actors

Despite being involved with IMPACT in an implementing capacity, or perhaps because they saw the process up close, Union Council (sub-district) managers told us they wanted ideation sessions of their own. These managers requested the opportunity to have their own IMPACT process of workshops and competitions where they could brainstorm ideas that would then have the potential for implementation.

Although managers and supervisors within the polio program worked in campaign planning, workforce management, and data consolidation, they were not given opportunities to shape policy. These (mostly) men at this middle level, between the frontline and higher-level officials, faced a lot of pressure to show progress and work overtime to present results. Some said that they were held accountable for subpar results stemming from poor policies that they had no power to change.

Many Union Council-level managers felt that their voices made little impact; one described his cadre of managers as “orphans.” Including them in the IMPACT process only as facilitators, and not as participants, may have exacerbated these feelings.

Managers told us that they appreciated how motivating the IMPACT process was for their frontline staff, but they also wanted a similar platform to propose solutions to issues they faced at their level. “We should also be given a chance,” one manager explained. “I want to share my ideas too, because we also face issues.”

Including sub-district level management and other frontline cadres of workers other than CHWs is something to consider in other IMPACT competitions. Hierarchy and power dynamics need to be considered. Holding separate competitions for middle-level managers and CHWs could be one potential way to avoid power dynamics playing out in unhelpful ways while also helping program planners draw on the deep experience and insights of sub-district level managers.

### The Value of Listening

The process of officials listening to, and implementing, CHW ideas was something new for the female CHWs who participated in the process. A forum like this was outside their experience, and at first CHWs did not believe it would live up to the description.

“Sorry, but we questioned the authenticity of this workshop at first,” one CHW commented. “We didn’t think it would really work.” She said, however, that she had been pleasantly surprised.

Across our interviews, workers told us that the IMPACT process made them feel like actors whose ideas mattered, rather than cogs in a vaccination and data collection machine. They were inspired by the feeling that their words could create tangible changes both in their work lives and toward the larger goal of achieving polio eradication—something they believed in.

“It made us realize how important we are,” one woman commented. “All this time, we thought our job was just limited to field work.”

## POLICY DESIGN FROM THE BOTTOM UP

The IMPACT process provided a structured and ultimately meaningful way for CHWs to contribute to the creation of improved program policy. For example, the process led to improved contracting and human resource processes for CHWs, and it resulted in improved outreach strategies to female religious leaders.^26^

IMPACT provided new platforms for voice for women at the bottom of health system structures, and the process was accepted by both lower- and higher-level supervisors and policymakers for at least two reasons. First, they appreciated the fact that the program was motivating for their frontline staff. Second, they also appreciated the insight into the policy changes they could make that would be most welcomed by CHWs providing vaccinations.

Through the IMPACT process, CHWs were able to give feedback to higher-level policymakers about what could improve the program, creating some degree of bottom-up programmatic input and design. This was acceptable to higher-level staff because they ultimately got to decide which CHW-generated policy change would actually be implemented. They did not cede control; rather, they were provided another mechanism for insight into their own program. CHWs understood that not all their suggestions would be implemented, but being invited to participate in improving policy design was deeply meaningful to them, giving their contributions to the program a new dimension of ownership and value.

In its competition format, the IMPACT process differs from other HCD processes. Although such a format is obviously not well suited for every design challenge, we believe it has several advantages. First, in a very hierarchical system—like many government health systems—it is a way to facilitate high-level buy-in of solutions generated by CHWs. Second, the competition format itself was motivating; it created energy, encouraged teamwork, and created a real sense of being heard and appreciated for short-listed teams.

With modest support for a few dedicated staff, it would be feasible to institutionalize a version of the IMPACT process within a range of medium- or large-scale CHW programs, providing a way to get solutions from frontline workers for a range of programmatic issues. Also, given the enthusiasm of lower-level managers for the process, it would be useful to explore giving other cadres the opportunity to participate in IMPACT as well.

There is ample reason to be circumspect about the potential of HCD processes like IMPACT. Design processes, like participatory processes in general, can simply become another mechanism for governing behavior within the same entrenched top-down development structures.^28^^,^^29^ Unlike radical design thinking paradigms,^30^ the IMPACT process did not revolutionize the oral polio vaccination program or lead to a new paradigm beyond the existing structures of vertical global health programming. Yet this process was meaningful to the women who participated in it. It gave low-income CHWs the chance to collaboratively build the program they were a part of and to have some input into the work structures that employed them. Some CHWs shared deep and sensitive issues that their supervisors had previously been unaware of, and some found joy in the process.

## Notes

**Peer Reviewed**

**First Published Online:** June 22 2026.
